# Ellagic acid protects dopamine neurons from rotenone‐induced neurotoxicity via activation of Nrf2 signalling

**DOI:** 10.1111/jcmm.15616

**Published:** 2020-07-12

**Authors:** Yi‐zheng Wei, Guo‐fu Zhu, Chang‐qing Zheng, Jing‐jie Li, Shuo Sheng, Dai‐di Li, Guo‐qing Wang, Feng Zhang

**Affiliations:** ^1^ Joint International Research Laboratory of Ethnomedicine of Ministry of Education and Key Laboratory of Basic Pharmacology of Ministry of Education Zunyi Medical University Zunyi China

**Keywords:** ellagic acid, neuroprotection, Nrf2, oxidative stress, Parkinson's disease

## Abstract

Parkinson's disease (PD) is the second most prevalent central nervous system (CNS) degenerative disease. Oxidative stress is one of key contributors to PD. Nuclear factor erythroid‐2‐related factor 2 (Nrf2) is considered to be a master regulator of many genes involved in anti‐oxidant stress to attenuate cell death. Therefore, activation of Nrf2 signalling provides an effective avenue to treat PD. Ellagic acid (EA), a natural polyphenolic contained in fruits and nuts, possesses amounts of pharmacological activities, such as anti‐oxidant stress and anti‐inflammation. Recent studies have confirmed EA could be used as a neuroprotective agent in neurodegenerative diseases. Here, mice subcutaneous injection of rotenone (ROT)‐induced DA neuronal damage was performed to investigate EA‐mediated neuroprotection. In addition, adult Nrf2 knockout mice and different cell cultures including MN9D‐enciched, MN9D‐BV‐2 and MN9D‐C6 cell co‐cultures were applied to explore the underlying mechanisms. Results demonstrated EA conferred neuroprotection against ROT‐induced DA neurotoxicity. Activation of Nrf2 signalling was involved in EA‐mediated DA neuroprotection, as evidenced by the following observations. First, EA activated Nrf2 signalling in ROT‐induced DA neuronal damage. Second, EA generated neuroprotection with the presence of astroglia and silence of Nrf2 in astroglia abolished EA‐mediated neuroprotection. Third, EA failed to produce DA neuroprotection in Nrf2 knockout mice. In conclusion, this study identified EA protected against DA neuronal loss via an Nrf2‐dependent manner.

## 1 INTRODUCTION

To date, the pathogenesis of Parkinson's disease (PD) is intangible.^1^ It has been verified that the increased and prolonged production of reactive oxygen species (ROS) plays critical roles in progress of PD.^2^ ROS compromises the balances between oxidant and anti‐oxidant systems.^3^ The anti‐oxidant defence system accounted for one of the underlying mechanisms that prevent oxidative stress‐induced cell damage.

Nuclear factor E2‐related factor 2 (Nrf2) regulates the physiological and pathophysiological processes of various diseases.^4^ Importantly, Nrf2 increases the gene expressions of endogenous anti‐oxidative enzymes, such as phase II detoxifying enzymes, quinone oxidoreductase 1 (NQO1) and NAD(P)H, heme oxygenase‐1 (HO‐1).^5^ A number of studies confirmed that Nrf2 is located and maintained in the cytoplasm in physiological conditions via a Kelch‐like ECH‐associated protein 1 (Keap1)‐dependent ubiquitination‐proteasomal degradation.^6^ Upon stimulated by oxidants or electrophiles, Nrf2 compromises Keap1/Nrf2 interactions and then Nrf2 dissociates from Keap1 complex and finally enters the nucleus. This is followed by binding to the anti‐oxidant response elements (ARE), a consensus gene sequence that encodes anti‐oxidant enzymes.^7^ Amounts of studies identified that Nrf2 signalling participates in PD pathogenesis.^8^ Activation of Nrf2 evoked dopamine (DA) neuroprotection and down‐regulation of Nrf2 rendered DA neurons susceptible to oxidative stress damage.^9^ Insufficient Nrf2 activation was highly involved in the progress of PD.^10^ Thus, potent Nrf2 signalling activation is thought to be a potential promising strategy for PD treatment.

Ellagic acid (2, 3, 7, 8‐tetrahydroxybenzopyrano [5, 4, 3‐cde] benzopyran‐5‐10‐ dione, EA) is located abundantly in plant extracts.^11^ EA presents a variety of pharmacological properties, such as anti‐inflammatory, cardioprotective and anti‐oxidant effects.^12^ Recently, several lines of evidence verified that EA conferred neuroprotection against aging, nerve dysfunction and neurodegeneration. In sporadic Alzheimer's disease animal model, EA generated neuroprotection and cognitive enhancement.^13^ In addition, EA protected against sodium arsenate‐induced neurotoxicity in rats.^14^ Moreover, EA was indicated to produce neuroprotection against ischemic stroke.^15^ However, the underlying mechanisms remain unclear.

In the present study, subcutaneous injection of rotenone (ROT)‐elicited PD mouse model was employed to investigate EA‐exerted DA neuroprotection. Furtherly, adult Nrf2 knockout mice and various cell cultures were applied to illuminate the role of Nrf2 signalling on EA‐mediated DA neuroprotection.

## 2 MATERIALS AND METHODS

### 2.1 Reagents

Ellagic acid (purity > 95%) and rotenone were obtained from Sigma Chemical (St. Louis, MO, USA). Anti‐tyrosine hydroxylase (TH, Catalog No. Ab113), haemeoxygenase‐1 (HO‐1, Catalog No. Ab68477), NADPH quinone oxidoreductase 1 (NQO1, Catalog No. ER1802‐86), Nrf2 (Catalog No. Ab31163), PCNA (Catalog No. 10205‐2‐AP), HSP90 (Catalog No. GB11284‐1) and β‐actin antibodies were bought from Abcam (Cambridge, MA, USA). The small interfering RNA (siRNA) against Nrf2 was purchased from Thermo Fisher Scientific (Waltham, MA, USA). SYBR green supermix (PCR) was obtained from Bio‐Rad Laboratories (CA, USA). RNAiso plus was purchased from Takara Biotech Co., Ltd. (Dalian, China).

### 2.2 Animals and treatment

Wild‐type (WT) C57BL/6J male mice and homozygous Nrf2 knockout (Nrf2^−/−^) male mice (22‐28 g, 10‐12 weeks) were purchased from the Nanjing University Biomedical Research Institute (Nanjing, China; Permit number: SCXK2015‐0001). All animals were kept in accordance with Chinese animal health and welfare norms. This study was approved by the Animal Care and Use Committee of Zunyi Medical University (Zunyi, China). WT and Nrf2 knockout mice were randomly divided into Control, EA (100 mg/kg), ROT (1 mg/kg), ROT + EA (20 mg/kg) and ROT + EA (100 mg/kg) groups. Mice were given subcutaneous injection of ROT (1 mg/kg) 6 times a week for consecutive 5 weeks. EA was administrated once a day for 5 weeks beginning 30 min before ROT injection.

### 2.3 Rotarod test

The rotarod test, in which animals must balance on the rod, was widely used to evaluate motor deficit in neurodegenerative disease models. Before the trial, all mice received rotarod training until they stayed on the stick at least for the cut‐off time. In the test, the started speed was 10 rpm and every 30 seconds increased 5 rpm until mice slid off the steps. Every mouse was tested 2 times a day, and the average duration of stay on the rod was recorded.

### 2.4 Open field test

Open field test was one of the most popular ethological tests to detect the anxiety‐like behaviour in animals. In the present study, mice were placed on the open field and each animal was located in a separate area and mice behavioural parameters were recorded during 5 minutes.^16^ Before each round of testing, the device should be cleaned with a 75% alcohol solution to eliminate odour interference. After the end of the experiment, the total distance of mice movement was calculated.

### 2.5 Immunohistochemistry staining and cell counting

Mice brains were cut into 7 microns cross‐sections on a paraffin slicer and attached to a glass slide. The brain slices were then dried and dewaxed. Fixed brain slices were separately treated with 3% hydrogen peroxide and 0.1 M citrate buffer and blocked with goat serum. Next, it was cultured overnight with an anti‐TH antibody at 4°C. Subsequently, brain sections were incubated with the secondary antibody working solution for 20 minutes followed by sections incubated with biotin for 15 minutes and developed with DAB developer. For morphological analysis, Olympus microscope (Tokyo, Japan) was applied to image TH‐positive neurons in SN. Finally, TH‐positive neurons were counted through counting the number of TH‐positive neuronal cell bodies blindly by two investigators and the results were averaged.

### 2.6 Western blotting

Total protein was extracted from mice midbrain by RIPA lysis solution. Nuclear and cytosolic fractions were extracted using a nuclear‐cell solute extraction kit (Solabio, Beijing, China). The protein concentrations were quantified by a BCA kit. Equal amounts of protein (10‐30 μg/lane) were separated on a 10% Bis‐Tris Nu‐PAGE gel. Then, proteins were transferred to a polyvinylidene fluoride (PVDF) membrane, blocked with 8% skim milk for 2.5 hours and placed in the primary antibody at 4°C overnight. The primary antibodies were as follows: TH (1:2000), Nrf2 (1:1000), HO‐1 (1:10 000), NQO1 (1:1000), PCNA (1:5000) and β‐actin (1:3000). Next, membranes were incubated with anti‐rabbit/mouse IgG secondary antibody at 1:2000 for 1 hours and detected with ECL substrate. The results were statistically analysed by quantitative analysis (Bio‐Rad, Hercules, CA, USA) software.

### 2.7 Real‐Time RT‐PCR Assay

Total RNA in the mice midbrain and cells were separately extracted by RNAiso plus reagent and purified with RNeasy Kits. The SYBR Green PCR Master Mix was used for real‐time PCR analysis. The primers used were designed by Sangon Biotech (Shanghai, China). The relative differences of the target genes were first normalized to β‐actin, and then calculated and expressed as a relative reduction or increase, setting the Control group at 100%.

### 2.8 Cell culture and treatment

BV‐2 microglia cell lines, C6 astroglia cell lines and MN9D DA neuron cell lines were obtained from the Chinese Academy of Sciences Cell Bank (Shanghai). All cultures were maintained in DMEM/F12 media supplemented with 10% foetal bovine serum and 1% antibiotics. Three kinds of cell lines were cultured at 37°C in a humidified atmosphere of 5% CO_2_ and 95% air. Cells were seeded at 1 × 10^6^/well in poly‐D‐lysinecoated 24‐well plates, respectively. Cells were treated with different concentrations of EA for 30 minutes followed by ROT (0.1 μmo/L) treatment for an additional 24 hours. Then, cells and culture medium were collected for later detection.

### 2.9 Nrf2‐siRNA transfection

C6 astroglia cell lines were seeds on 24‐well plates for 24 hours and then transfected with Nrf2‐siRNA (50 nmol/L) or control‐siRNA (50 nmol/L) for 6 hours using siRNA transfection reagent (Thermo Fisher Scientific) using the manufacturer's protocol. The knockdown efficiency was evaluated by Western blotting and real‐time RT‐PCR. After 6 hours of transfection, the transfection solution was removed and cells were treated with EA for 24 hours followed by ROT administration for 24 hours. Afterwards, C6 cells were collected to detect the corresponding indicators.

### 2.10 Reconstituted neuron‐microglia and neuron‐astroroglia co‐cultures using transwells

BV‐2 cells or C6 cells were cultured in the upper chamber of transwell inserts for 24 hours. The lower chambers contained MN9D cells were also cultured for 24 hours. Then, BV‐2 or C6 cells in transwells were transferred into MN9D cells, and the reconstituted neuron‐microglia (MN9D‐BV‐2) and neuron‐astroglia (MN9D‐C6) co‐cultures were established. These co‐cultures were treated with EA for 30 minutes followed by ROT application for 24 hours. For MN9D‐C6 co‐cultures, the C6 cells in transwells were processed by Nrf2‐siRNA for 6 hours. Then, the MN9D cells in lower chambers were cultured for 24 hours. Next, C6 cells were rinsed with fresh medium and transferred into MN9D cultures. The reconstituted neuron‐astroglia co‐cultures conditionally silencing astroglia Nrf2 were established. Finally, cultures were treated with EA for 30 minutes followed by ROT treatment for 24 hours.^17^, ^18^ DA neuronal damage was assessed by TH‐positive neuron staining and TH protein expression.

### 2.11 Cell viability assay

After EA treatment for 24 hours, the upper chambers were moved and the lower chambers were incubated with MTT solution (5 mg/mL) for 4 hours. Dimethyl sulfoxide (DMSO) was added to each well to determine cell viability. Absorbance values were measured at 490 nm.

### 2.12 Immunocytochemical staining

DA neurons were recognized with an anti‐TH antibody. Cells were fixed with 4% paraformaldehyde followed by permeabilization using 0.03% Triton X‐100 and closed with goat serum. DA neurons were labelled with an anti‐TH (1:300) antibody at 4°C overnight and then incubated with an anti‐rabbit‐IgG (1:2000) antibody for 1 hours. TH‐positive neuron numbers were calculated from four representative areas per well of the 24‐well plate. In each condition, three wells were used for cell counting.

### 2.13 Statistical analysis

Data analyses were performed as mean ± SEM. Statistical comparisons were analysed using the SPSS statistical software by one‐way ANOVA. Then, the Bonferroni's *post hoc* test was performed for all pairwise comparisons among means. *P* < 0.05 was considered as statistically significant.

## 3 RESULTS

### 3.1 EA protected against ROT‐induced DA neuronal damage

**FIGURE 1:**
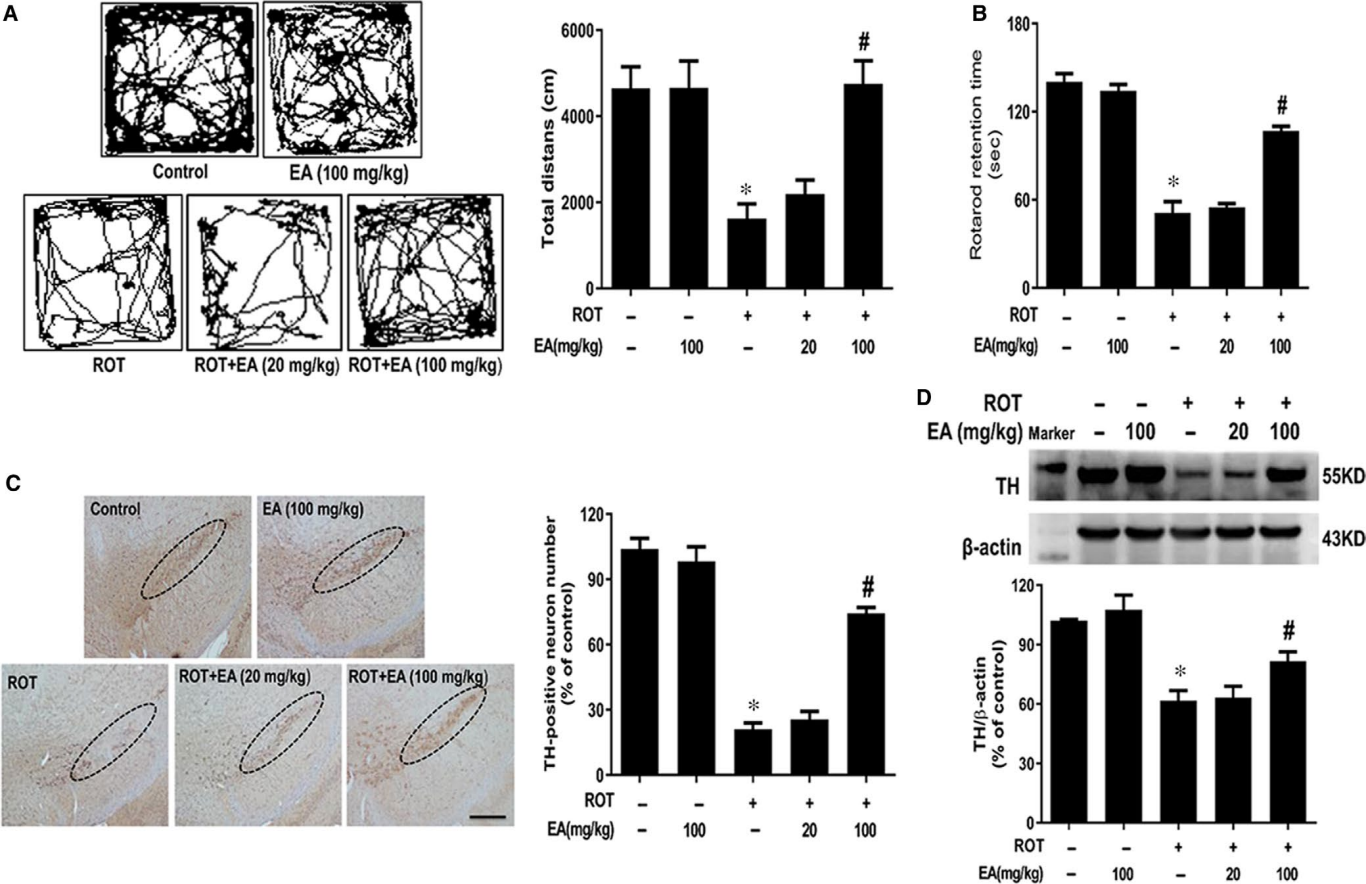
EA ameliorated ROT‐induced DA neuronal damage. Male wild‐type mice received subcutaneous injection of ROT (1 mg/kg) six times a week for consecutive five weeks. EA were daily given by intragastric administration for consecutive another five weeks. Open field test and rotarod test were performed to evaluate animal behaviour changes (A and B). TH‐positive neuron number was counted after midbrain sections immunohistochemistry staining (C). Scale bar = 200 μm. TH protein expression was tested by Western blot assay (D). Results were represented as mean ± SEM from 6 mice. *P < 0.05 compared with the control group, ^#^ *P* < 0.05 compared with ROT‐treated group

To explore the neuroprotective effects of EA on PD, we established ROT‐induced DA neuronal lesion in mice and determined whether EA could attenuate ROT‐induced DA neurotoxicity. First, we examined behaviour changes by open field and rotarod tests. As shown in Figure 1A and B, ROT caused an apparent reduction in total movement distance and the time mice stayed on the rod (Figure S2). EA exhibited a significant improvement in locomotor dysfunction. Furthermore, ROT decreased TH‐positive neuronal number and TH protein expression and EA ameliorated ROT‐induced DA neuronal damage (Figure 1C and D).

### 3.2 EA activated Nrf2 signalling pathway in vivo

**FIGURE 2:**
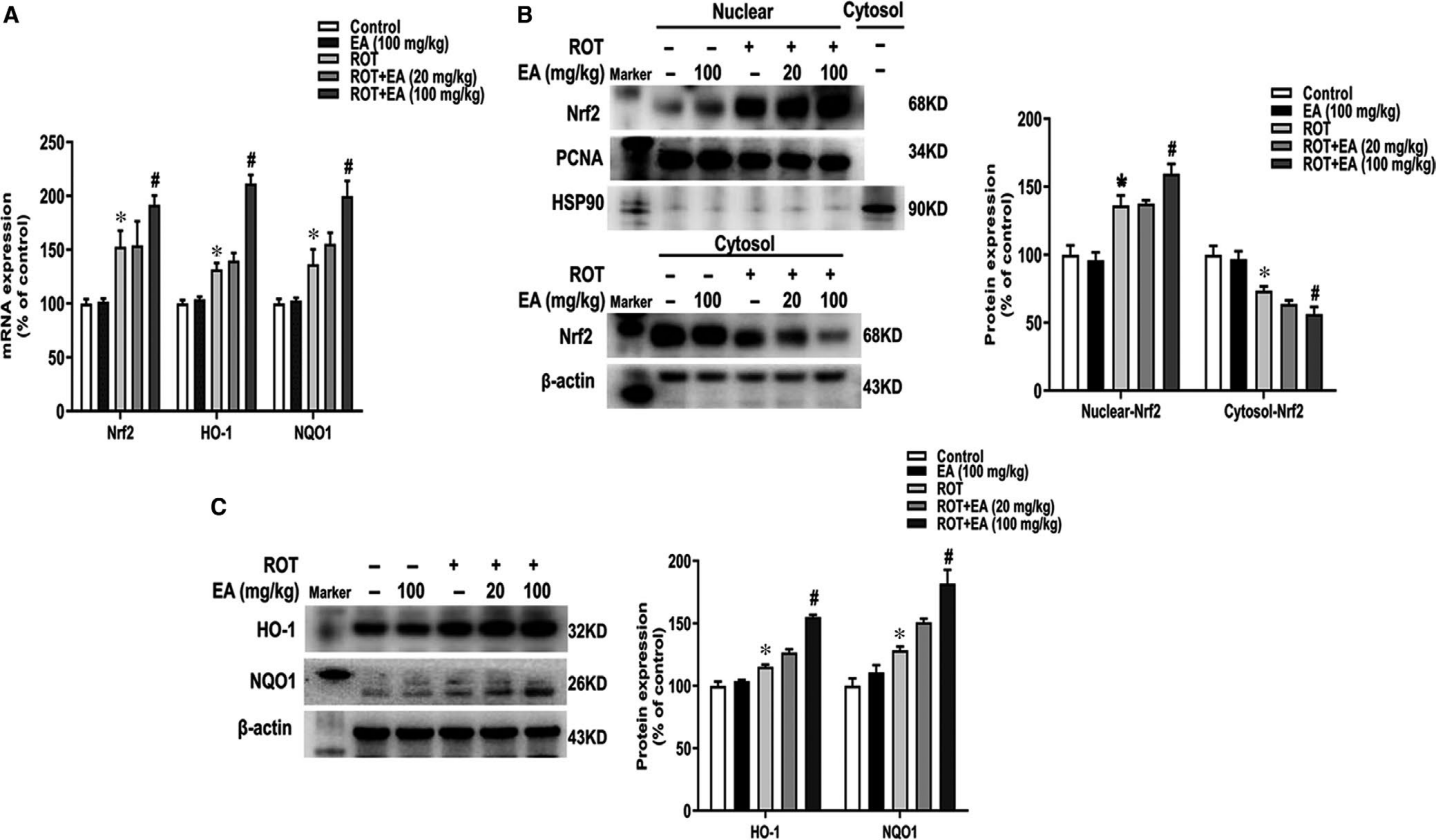
EA activated Nrf2 signalling pathway in vivo. The mRNA expressions of Nrf2, HO‐1 and NQO1 in mice midbrain were determined by real‐time RT‐PCR (A). The protein expressions of nuclear Nrf2 and PCNA, cytosol Nrf2 and HSP90, whole cell HO‐1 and NQO1 were measured by Western blot assay (B and C). Results were mean ± SEM from 6 mice. *P < 0.05 compared with the control group, ^#^ *P* < 0.05 compared with ROT‐treated group

To determine whether EA could activate Nrf2 signalling pathway, the expressions of Nrf2, HO‐1 and NQO1 were detected by real‐time RT‐PCR and Western blot assay. As shown in Figure 2A, the mRNA expressions of Nrf2, HO‐1 and NQO1 were increased in the ROT and EA groups, in which EA induced higher mRNA expressions of these genes than ROT. To further determine the effects of EA on Nrf2 distribution, the cytosolic components and nuclear fractions were collected, respectively. The cytosol marker HSP90 was used to verify the purity of nuclear protein, and the nuclear fraction was not contaminated by cytosol proteins as confirmed by the absence of HSP90. As shown in Figure 2B, compared with control group, ROT activated Nrf2 and induced Nrf2 to transfer from cytosol to nucleus. Compared with ROT group, the increase in translocation of Nrf2 from cytosol to nucleus was more pronounced in EA group. Meanwhile, the higher protein expressions of HO‐1 and NQO1 in ROT + EA group were indicated than those in ROT group (Figure 2C).

### 3.3 EA targeted astroglia to protect from ROT‐induced DA neuronal damage

**FIGURE 3:**
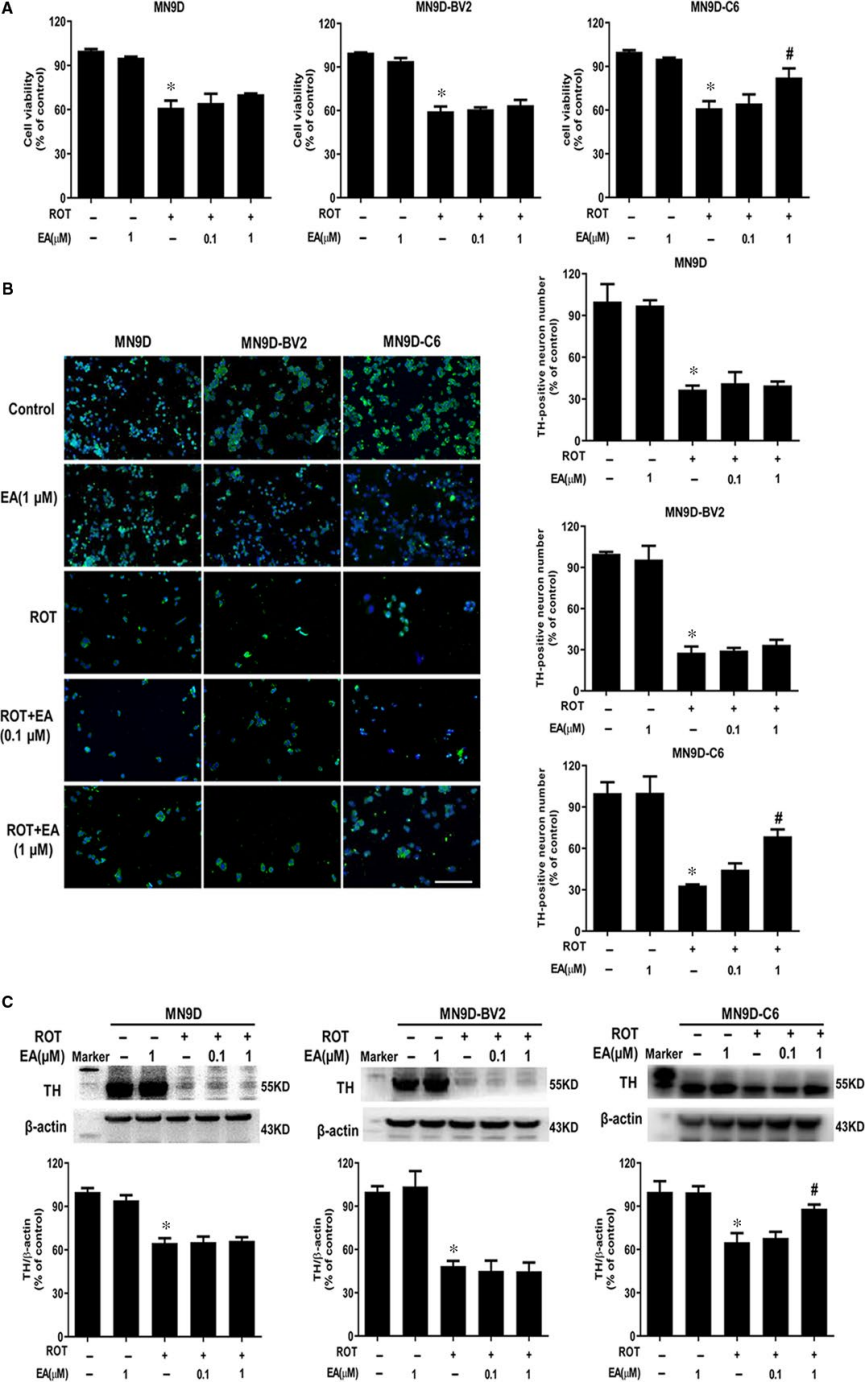
EA targeted astroglia to protect against ROT‐induced DA neuronal damage. MN9D‐enriched cultures, MN9D‐BV2 and MN9D‐C6 co‐cultures were cultured for 24 h, respectively. EA was pre‐treated for 30 min and then incubated with ROT for another 24 h. The neurotoxicity of ROT and EA on cell viability in MN9D, MN9D‐BV2 and MN9D‐C6 cultures was determined by MTT assay (A). DA neuron number in these 3 cultures was counted after TH‐positive neurons immunofluorescence staining (B). Scale bar = 100 μm. TH protein expression in 3 cell systems was detected by Western blot assay (C). Results were mean ± SEM from three independent experiments. *P < 0.05 compared with the control cultures, ^#^ *P* < 0.05 compared with ROT‐treated cultures

To further explore which cell type EA targeted to produce DA neuroprotection, three cell culture systems including MN9D‐enriched, MN9D‐BV2 and MN9D‐C6 co‐cultures were performed using transwells. Cultures were treated with EA for 30 minutes followed by ROT treatment for 24 hours. As shown in MTT assay (Figure 3A), EA‐mediated neuroprotection was indicated in MN9D‐C6 co‐culture system but not in either MN9D‐enriched or MN9D‐BV2 co‐cultures (Figure S2). Consistent with MTT assay, EA decreased ROT‐induced LDH release in MN9D‐C6 co‐culture but not in MN9D‐enriched or MN9D‐BV2 co‐cultures (Figure S1). Moreover, from TH‐positive neuronal immunofluorescence staining and TH protein detection assays shown in Figure 3B and C, in MN9D‐C6 co‐culture system, EA attenuated ROT‐induced decrease of TH‐positive neuronal number and TH protein expression. However, in MN9D‐enriched cultures and MN9D‐BV2 co‐cultures, this neuroprotection disappeared. These phenomenon demonstrated astroglia participated in EA‐elicited neuroprotection.

### 3.4 EA activated Nrf2 signalling pathway in vitro

**FIGURE 4:**
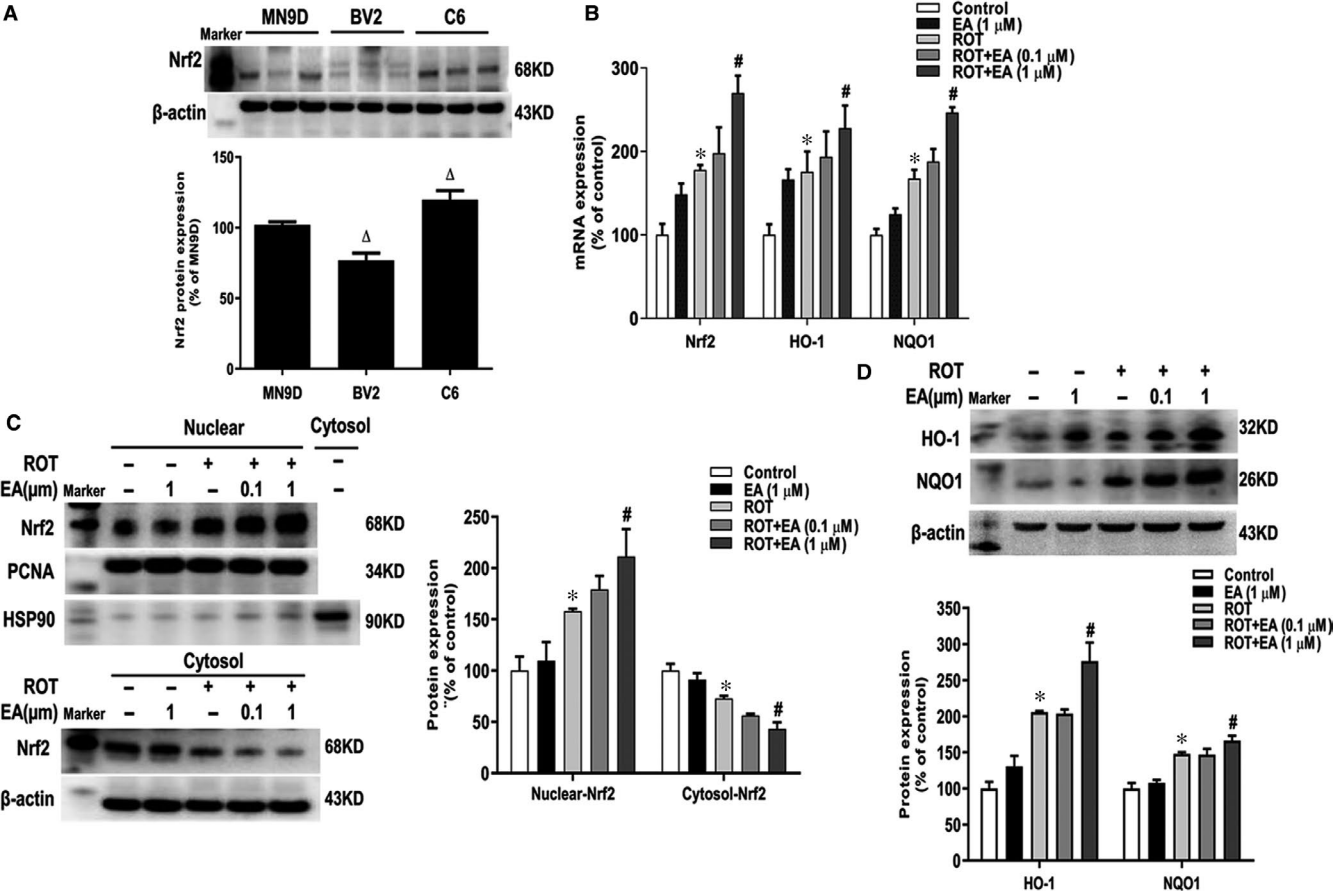
EA activated Nrf2 signalling pathway in vitro. Protein expression of Nrf2 in MN9D, BV2 and C6 cultures was detected by Western blot assay (A). C6 cells were pre‐treated with EA (0.1 and 1 μM) for 30 min and then stimulated by ROT (0.1 μM) for 24 h. The mRNA expressions of Nrf2, HO‐1 and NQO1 were determined by real‐time RT‐PCR (B). The protein expressions of nuclear Nrf2 and PCNA, cytosol Nrf2 and HSP90, whole cell HO‐1 and NQO1 were measured by Western blot assay (C and D). Results were mean ± SEM from three independent experiments performed in triplicate. *P < 0.05 compared with the control cultures, ^#^ *P* < 0.05 compared with ROT‐treated cultures, ^Δ^ *p* < 0.05 compared with MN9D cell cultures

To confirm the activation of Nrf2 signalling induced by EA treatment in which cell type, the expressions of Nrf2 signalling in MN9D, BV2 and C6 cells were detected. As shown in Figure 4A, Nrf2 protein expression mainly concentrated in C6 cells. To further determine whether EA activated Nrf2 signalling in astroglia, the mRNA and protein expressions of Nrf2 signalling in C6 cells were examined. As shown in Figure 4B, Nrf2, HO‐1 and NQO1 mRNA levels were up‐regulated in ROT and ROT + EA treatment groups, whereas EA enhanced these genes mRNA expressions compared with ROT administration. In addition, Western blot assay showed the translocation of Nrf2 from cytoplasm to the nucleus was more obvious after EA treatment that that in ROT group (Figure 4C and S3). Also, EA induced the higher protein expressions of HO‐1 and NQO1 than ROT (Figure 4D).

### 3.5 EA protected DA neurons by the activation of Nrf2 signalling

**FIGURE 5:**
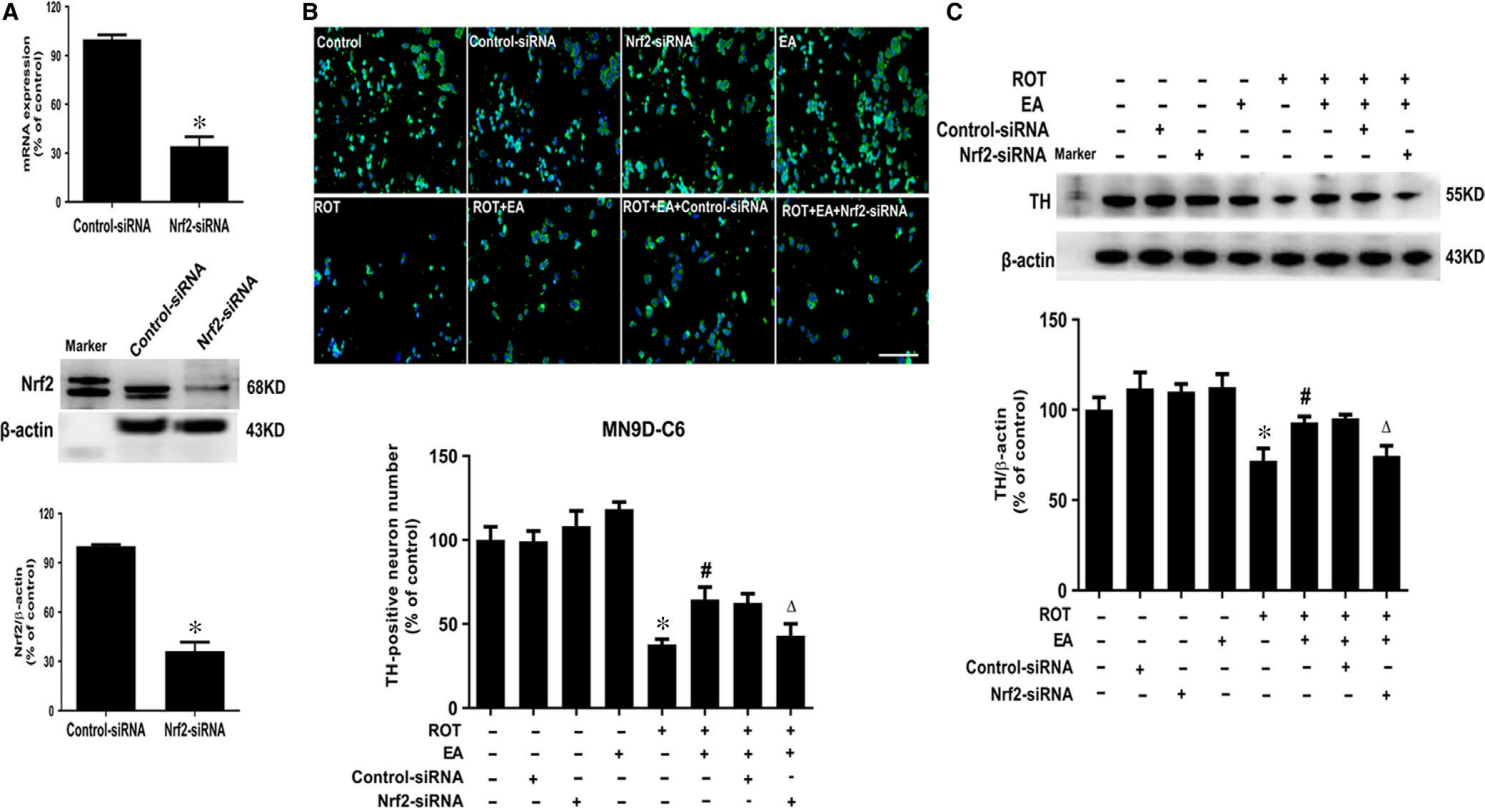
Role of Nrf2 in EA‐mediated DA neuroprotection. C6 cells were treated with Nrf2 siRNA (50 nmol/L). After 6 h of transfection, the transfection solution was removed and cells were rinsed with PBS. The silence efficiency was validated by real‐time RT‐PCR and Western blotting (A). Then, C6 cells conditionally silencing Nrf2 were seeded in transwells upper chamber followed by MN9D cells seeded in the lower chamber to establish MD9D‐C6 co‐cultures and then treated with EA for 30 min and ROT for another 24 h. DA neuronal damage was determined by TH‐positive neuronal number counting (B) and TH protein expression detection (C). Scale bar = 100 μm. Results were mean ± SEM from three independent experiments performed in triplicate. *P < 0.05 compared with the control cultures, ^#^ *P* < 0.05 compared with ROT‐treated cultures, ^△^ *P* < 0.05 compared with ROT + EA cultures

To explore the role of Nrf2 in EA‐mediated DA neuroprotection, Nrf2 siRNA in vitro and Nrf2 knockout mice were performed. As shown in Figure 5A, Nrf2 gene and protein levels were down‐regulated in C6 cells transfected with Nrf2‐siRNA with the silence ratio of 70%. Furtherly, in TH‐positive neuronal counting and TH protein expression assays, EA‐generated DA neuroprotection from ROT‐induced neurotoxicity was neutralized by Nrf2‐siRNA application (Figure 5B and C).

**FIGURE 6:**
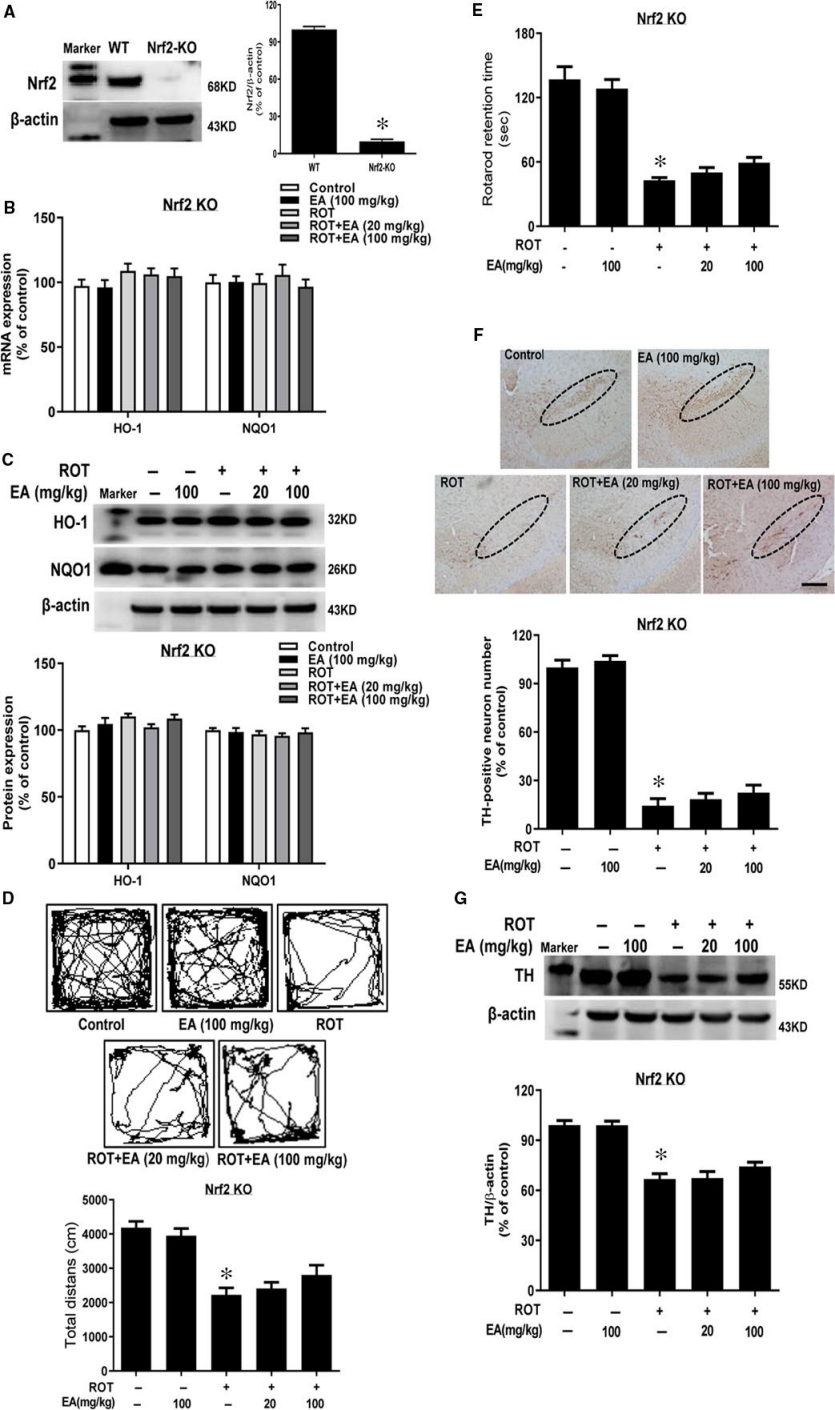
EA‐elicited neuroprotection was abolished in Nrf2 knockout mice. Nrf2 knockout mice received subcutaneous injection of ROT (1 mg/kg) six times a week for consecutive five weeks followed by intragastric administration of EA for another five weeks. Western blotting was used to test knockout efficiency in Nrf2 knockout mice (A). The mRNA and protein expressions of HO‐1 and NQO1 in mice midbrain were detected via real‐time RT‐PCR (B) and Western blot assays (C). Mice behaviour changes were assessed by open field (D) and rotarod tests (E). Brain sections were immunostained, and the number of TH‐positive neurons in mice SN was counted (F). The ‘ellipse’ presented the area of SN. Scale bar = 200 μm. TH protein expression in SN was measured by Western blot assay (G). Results were mean ± SEM from 6 mice. *P < 0.05 compared with the control group

Next, Nrf2 knockout mice were employed to confirm EA evoked neuroprotection via an Nrf2‐dependent manner in vivo. First, the knockout efficiency was verified by Nrf2 protein level detection (Figure 6A). Then, there was no significant difference of the genes and proteins expressions of HO‐1 and NQO1 in the midbrain of Nrf2 knockout mice after EA treatment (Figure 6B and C). In addition, EA failed to improve the time mice stayed on rod and locomotor distance in Nrf2 knockout mice (Figure 6D and E). Meanwhile, TH‐positive neuronal counting and TH protein expression detection showed EA‐mediated DA neuroprotection was abolished in Nrf2 knockout mice (Figure 6F and G). Collectively, these results demonstrated EA protected DA neurons through the activation of astroglial Nrf2 signalling.

## 4 DISCUSSION

The present study aimed at investigating the neuroprotective actions of EA on ROT‐induced DA neuronal damage and the underlying mechanisms as well. Results demonstrated that EA conferred neuroprotection against ROT‐induced DA neurotoxicity. Activation of Nrf2 signalling was involved in EA‐mediated DA neuroprotection, as evidenced by the following observations. First, EA activated Nrf2 signalling pathway in ROT‐induced DA neuronal damage. Second, EA generated neuroprotection with the presence of astroglia and silence of Nrf2 in astroglia abolished EA‐mediated neuroprotection. Third, EA failed to produce DA neuroprotection in Nrf2 knockout mice. Together, these results suggested EA‐generated DA neuroprotection might be attributable to the activation of Nrf2 signalling.

Oxidative stress has been considered as the central pathological event in the development of PD.^19^, ^20^ For survival and development of neurons, the maintenance of redox homeostasis in the CNS was indispensable.^21^ Nrf2 was an oxidative defence transcription factor which mitigated the toxic neuronal effects of parkinsonian agents, such as ROT, 1‐methyl‐4‐phenyl‐1,2,3,6‐tetrahydropyridine (MPTP) and 6‐hydroxydopamine (6‐OHDA) in vivo and *in vitro*.^22^ Here, this study found that EA protected DA neurons activated Nrf2 signalling pathway and initiated the Nrf2 stressor mechanism for its transfer from cytosol to the nucleus (Figures 1 and 2). Further study indicated that EA‐elicited neuroprotection were abolished in Nrf2 knockout mice (Figure 6). These results suggested that EA‐enhanced DA neuronal survival was dependent on Nrf2 activation.

Encouraged by the activation of Nrf2 signalling by EA, we further investigated Nrf2 signalling in which cell type participated in EA‐mediated neuroprotection. It is interesting to note that EA targeted astroglia to produce neuroprotection as this protection was just indicated in the presence of astroglia (Figure 3). Astroglia is the most abundant cell type in the brain.^23^ They interacted with neurons in various aspects of signalling transmission, immune and oxidative defence, ion and water homeostasis, metabolite supply and neuroprotection.^24^ In neurological disorders, astroglia themselves formed a defence barrier and promoted neuroprotection by producing anti‐oxidants against inflammation, especially for excessive extracellular release of the excitatory neurotransmitter glutamate.^25^ Stimulation of sulforaphane also prolonged Nrf2‐mediated gene expression in astrocytes and prevented neuronal damage caused by superoxide.^26^ In addition, besides anti‐oxidants, astrocytes could release neurotrophic factors, such as BDNF and GDNF‐mediated activation of the Nrf2 pathway in astrocytes, which increased the survival of DA neurons.^27^ Recent research indicated that Nrf2‐ARE pathway was preferentially activated in astrocytes, HO‐1 and NQO1were strongly expressed in astrocytes, sometime microglia, with more infrequent expression in neurons.^28^, ^29^ Nevertheless, Pharmacological activation and overexpression of Nrf2 in astrocytes also clearly demonstrated its therapeutic potential for PD. In the mouse model of α‐synuclein mutation, the specific overexpression of astrocyte Nrf2 was regulated, which improved the movement and non‐motor dysfunction of the whole CNS, and delayed the process of α‐synuclein aggregation.^30^ Also, Nrf2 overexpressed in astrocytes kept mice from mutated α‐synuclein to protect DA neurons.^31^ Additionally, transplantation of astrocytes overexpressing Nrf2 into mouse striatum protected from DA neuronal loss.^32^ Therefore, this was an important strategy for the support of neuroprotective functions of astrocytes to enhance neuronal survival and improve PD symptoms. In this study, there was no direct contact between astroglia and neurons via neuron‐astroglia reconstituted cultures using transwells. Here, we speculated that EA might increase astroglial neurotrophic factors release via enhancing Nrf2 activation. The neurotrophic factors have been confirmed to be the key factors in promoting the neuronal survival, growth and differentiation. The absence and depletion of neurotrophic factors are revealed to be closely related to the pathogenesis of PD. Furthermore, dopamine released in the extracellular space from DA neurons seemed to induce oxidative stress. Also, such DA induced the activation of Nrf2 signalling in astroglia, which in turn released the reduced form of glutathione (GSH) that erased ROS generated by DA.^33^ This could be one of the mechanisms underlying EA‐enhanced astroglial neuroprotective function against ROT‐induced neurotoxicity in the present study.

A large amount of evidence suggested Nrf2 activators could protect neurons and decrease the accumulation of aberrant proteins in vitro and in vivo in different neurodegenerative mouse models. In clinic, melatonin was found to activate Nrf2 to rescue hippocampal bioenergetics and improve cognitive function.^34^ At present, the clinical trials on Nrf2 activators for the treatment of neurodegenerative diseases were increasing.^35^ For example, pinostrobin could release HO‐1 through Nrf2 activation to alleviate MPTP‐induced neuronal loss in SN, and relieved zebra fish behavioural deficit disorder, and provided potential neuroprotective effects in vivo.^25^ Moreover, Glaucocalyxin B was discerned to inhibit TLR/NF‐κB pathway activation and induce Nrf2/HO‐1 activation and further produce neuroprotection.^36^ Also, dimethyl fumarate enhanced Nrf2 activation, thereby playing an important role in the defence of MPTP‐induced loss of DA neurons in the SN.^37^ In short, new Nrf2‐based treatments might be considered as a viable strategy for future potential. Thus, this study found EA exhibited a promising therapeutic efficacy for PD via activating Nrf2 signalling pathway. These findings suggested that modulation of Nrf2 signalling by potential candidates, such as EA, might be beneficial for PD treatment. Despite this optimistic perspective for future administration of EA potential treatment for PD, most of the findings were derived from experimental models and in vitro data and therefore need be rigorously corroborated in clinical trials in future.

## 5 CONCLUSION

This study demonstrated that EA protected DA neurons from ROT‐induced neurotoxicity by activating Nrf2 signalling. These findings suggested EA might be an exciting option for treating PD.

## CONFLICT OF INTERESTS

The authors declared no conflicts of interests.

## AUTHOR CONTRIBUTION

**Yi‐zheng Wei:** Data curation (equal); Writing‐original draft (equal); Writing‐review & editing (equal). **Guo‐fu Zhu:** Investigation (equal); Methodology (equal). **Chang‐qing Zheng:** Investigation (equal); Methodology (equal). **Jing‐jie Li:** Investigation (equal); Supervision (equal). **Shuo Sheng:** Investigation (equal); Supervision (equal). **Dai‐Di Li:** Investigation (equal); Supervision (equal). **Guo‐Qing Wang:** Writing‐original draft (equal); Writing‐review & editing (equal). **Feng Zhang:** Conceptualization (equal); Funding acquisition (equal); Writing‐original draft (equal); Writing‐review & editing (equal).

## ETHICAL APPROVAL

All experimental procedures were carried out in accordance with Chinese Guidelines of Animal Care and Welfare and this study received an approval from the Animal Care and Use Committee of Zunyi Medical University (Zunyi, China).

## Supporting information

Fig S1

Fig S2

Fig S3

## Data Availability

The data that support the findings of this study are available from the corresponding author upon reasonable request.

## References

[1] Bergstrom P , Andersson HC , Gao Y , et al. Fyn Kinase Regulates Microglial Neuroinflammatory Responses in Cell Culture and Animal Models of Parkinson's Disease. Neuropharmacology. 2011;60(2‐3):343‐353.20888844

[2] Maiti P , Manna J , Dunbar GL . Current understanding of the molecular mechanisms in Parkinson's disease: Targets for potential treatments. Transl Neurodegen. 2017;6:28. 10.1186/s40035-017-0099-z PMC5655877 29090092

[3] Tansey MG , McCoy MK , Frank‐Cannon TC . Neuroinflammatory mechanisms in Parkinson's disease: potential environmental triggers, pathways, and targets for early therapeutic intervention. Exp Neurol. 2007;208(1):1‐25. 17720159 10.1016/j.expneurol.2007.07.004 PMC3707134

[4] AlDakheel A , Kalia LV , Lang AE . Pathogenesis‐targeted, disease‐modifying therapies in Parkinson disease. Neurotherapeutics. 2014;11(1):6‐23. 24085420 10.1007/s13311-013-0218-1 PMC3899477

[5] Yan J , Li J , Zhang L , et al. Nrf2 protects against acute lung injury and inflammation by modulating TLR4 and Akt signaling. Free Radic Biol Med. 2018;121:78‐85. 29678610 10.1016/j.freeradbiomed.2018.04.557

[6] Ruiz S , Pergola PE , Zager RA , Vaziri ND . Targeting the transcription factor Nrf2 to ameliorate oxidative stress and inflammation in chronic kidney disease. Kidney Int. 2013;83(6):1029‐1041. 23325084 10.1038/ki.2012.439 PMC3633725

[7] Zhao Y , Song W , Wang Z , et al. Resveratrol attenuates testicular apoptosis in type 1 diabetic mice: Role of Akt‐mediated Nrf2 activation and p62‐dependent Keap1 degradation. Redox Biol. 2018;14:609‐617. 29154192 10.1016/j.redox.2017.11.007 PMC5975057

[8] Dheen ST , Jun Y , Yan Z , et al. Retinoic acid inhibits expression of TNF‐alpha and iNOS in activated rat microglia. Glia. 2005;50(1):21‐31. 15602748 10.1002/glia.20153

[9] Lastres‐Becker I , Garcia‐Yague AJ , Scannevin RH , et al. Repurposing the NRF2 Activator Dimethyl Fumarate as Therapy Against Synucleinopathy in Parkinson's Disease. Antioxid Redox Signal. 2016;25(2):61‐77. 27009601 10.1089/ars.2015.6549 PMC4943471

[10] Vargas MR , Pehar M , Cassina P , et al. Increased glutathione biosynthesis by Nrf2 activation in astrocytes prevents p75NTR‐dependent motor neuron apoptosis. J Neurochem. 2006;97(3):687‐696. 16524372 10.1111/j.1471-4159.2006.03742.x

[11] Neymotin A , Calingasan NY , Wille E , et al. Neuroprotective effect of Nrf2/ARE activators, CDDO ethylamide and CDDO trifluoroethylamide, in a mouse model of amyotrophic lateral sclerosis. Free Radic Biol Med. 2011;51(1):88‐96. 21457778 10.1016/j.freeradbiomed.2011.03.027 PMC3109235

[12] Firdaus F , Zafeer MF , Anis E , et al. Ellagic acid attenuates arsenic induced neuro‐inflammation and mitochondrial dysfunction associated apoptosis. Toxicol. Rep. 2018;5:411‐417. 29854611 10.1016/j.toxrep.2018.02.017 PMC5978009

[13] Jha AB , Panchal SS , Shah A . Ellagic acid: Insights into its neuroprotective and cognitive enhancement effects in sporadic Alzheimer's disease. Pharmacol Biochem Behav. 2018;175:33‐46. 30171934 10.1016/j.pbb.2018.08.007

[14] Goudarzi M , Amiri S , Nesari A , et al. The possible neuroprotective effect of ellagic acid on sodium arsenate‐induced neurotoxicity in rats. Life Sci. 2018;198:38‐45. 29455002 10.1016/j.lfs.2018.02.022

[15] Liu QS , Deng R , Li S , et al. Ellagic acid protects against neuron damage in ischemic stroke through regulating the ratio of Bcl‐2/Bax expression. Appl. Physiol. Nutr. Metabol. 2017;42(8):855‐860. 10.1139/apnm-2016-0651 28388366

[16] Kraeuter AK , Guest PC , Sarnyai Z . The open field test for measuring locomotor activity and anxiety‐like behavior. Method Mol. Biol. 2019;1916:99‐103. 10.1007/978-1-4939-8994-2_9 30535687

[17] Bates AM , Fischer CL , Abhyankar VP , et al. Matrix metalloproteinase response of dendritic cell, gingival epithelial keratinocyte, and T‐Cell transwell co‐cultures treated with *Porphyromonas gingivalis* Hemagglutinin‐B. Int J Mol Sci. 2018;19(12):3923. 10.3390/ijms19123923 PMC6321455 30544510

[18] Chen SH , Oyarzabal EA , Sung YF , et al. Microglial regulation of immunological and neuroprotective functions of astroglia. Glia. 2015;63(1):118‐131. 25130274 10.1002/glia.22738 PMC4237670

[19] Surendran S , Rajasankar S . Parkinson's disease: oxidative stress and therapeutic approaches. Neurol. Sci. 2010;31(5):531‐540. 20221655 10.1007/s10072-010-0245-1

[20] Guo JD , Zhao X , Li Y , et al. Damage to dopaminergic neurons by oxidative stress in Parkinson's disease (Review). Int J Mol Med. 2018;41(4):1817‐1825. 29393357 10.3892/ijmm.2018.3406

[21] Xicoy H , Wieringa B , Martens GJ . The SH‐SY5Y cell line in Parkinson's disease research: a systematic review. Mol Neurodegener. 2017;12(1):10. 28118852 10.1186/s13024-017-0149-0 PMC5259880

[22] Huang B , Liu J , Meng T , et al. Polydatin Prevents Lipopolysaccharide (LPS)‐Induced Parkinson's Disease via Regulation of the AKT/GSK3beta‐Nrf2/NF‐kappaB Signaling Axis. Front Immunol. 2018;9:2527. 30455692 10.3389/fimmu.2018.02527 PMC6230593

[23] Gorshkov K , Aguisanda F , Thorne N , Zheng W . Astrocytes as targets for drug discovery. Drug Discovery Today. 2018;23(3):673‐680. 29317338 10.1016/j.drudis.2018.01.011 PMC5937927

[24] di Val R , Cervo P , Romanov RA , et al. Induction of functional dopamine neurons from human astrocytes in vitro and mouse astrocytes in a Parkinson's disease model. Nat Biotechnol. 2017;35(5):444‐452. 28398344 10.1038/nbt.3835

[25] Liu B , Teschemacher AG , Kasparov S . Astroglia as a cellular target for neuroprotection and treatment of neuro‐psychiatric disorders. Glia. 2017;65(8):1205‐1226. 28300322 10.1002/glia.23136 PMC5669250

[26] Bergstrom P , Andersson HC , Gao Y , et al. Repeated transient sulforaphane stimulation in astrocytes leads to prolonged Nrf2‐mediated gene expression and protection from superoxide‐induced damage. Neuropharmacology. 2011;60(2–3):343‐353. 20888844 10.1016/j.neuropharm.2010.09.023

[27] Ishii T , Warabi E , Mann GE . Circadian control of BDNF‐mediated Nrf2 activation in astrocytes protects dopaminergic neurons from ferroptosis. Free Radic Biol Med. 2019;133:169‐178. 30189266 10.1016/j.freeradbiomed.2018.09.002

[28] Joe EH , Choi DJ , An J , et al. Astrocytes, Microglia, and Parkinson's disease. Experimental neurobiology. 2018;27(2):77‐87. 29731673 10.5607/en.2018.27.2.77 PMC5934545

[29] de Freitas SM , Pruccoli L , Morroni F , et al. The Keap1/Nrf2‐ARE Pathway as a Pharmacological Target for Chalcones. Molecules. 2018;23(7):1803. 10.3390/molecules23071803 PMC6100069 30037040

[30] Gan L , Vargas MR , Johnson DA , Johnson JA . Astrocyte‐specific overexpression of Nrf2 delays motor pathology and synuclein aggregation throughout the CNS in the alpha‐synuclein mutant (A53T) mouse model. J Neurosci. 2012;32(49):17775‐17787. 23223297 10.1523/JNEUROSCI.3049-12.2012 PMC3539799

[31] Chen PC , Vargas MR , Pani AK , et al. Nrf2‐mediated neuroprotection in the MPTP mouse model of Parkinson's disease: Critical role for the astrocyte. Proc Natl Acad Sci USA. 2009;106(8):2933‐2938. 19196989 10.1073/pnas.0813361106 PMC2650368

[32] Liddell JR . Are astrocytes the predominant cell type for activation of Nrf2 in aging and neurodegeneration? Antioxidants. 2017;6(3):65. 10.3390/antiox6030065 PMC5618093 28820437

[33] Mashima K , Takahashi S , Minami K , et al. Neuroprotective role of Astroglia in Parkinson disease by reducing oxidative stress through dopamine‐induced activation of pentose‐phosphate pathway. ASN Neuro. 2018;10:1759091418775562 29768946 10.1177/1759091418775562 PMC5960859

[34] Chen LY , Renn TY , Liao WC , et al. Melatonin successfully rescues hippocampal bioenergetics and improves cognitive function following drug intoxication by promoting Nrf2‐ARE signaling activity. J Pineal Res. 2017;63(2):e12417. 10.1111/jpi.12417 28480587

[35] Houghton CA , Fassett RG , Coombes JS . Sulforaphane and other nutrigenomic NRF2 activators: can the clinician's expectation be matched by the reality? Oxidat Med Cell Long. 2016;2016:7857186. 10.1155/2016/7857186 PMC4736808 26881038

[36] Xu W , Zheng D , Liu Y , et al. Glaucocalyxin B Alleviates Lipopolysaccharide‐Induced Parkinson's Disease by Inhibiting TLR/NF‐kappaB and Activating Nrf2/HO‐1 Pathway. Cell Physiol Biochem. 2017;44(6):2091‐2104. 29241205 10.1159/000485947

[37] Campolo M , Casili G , Biundo F , et al. The neuroprotective effect of Dimethyl Fumarate in an MPTP‐mouse model of parkinson's disease: involvement of reactive oxygen species/nuclear Factor‐kappaB/nuclear transcription factor related to NF‐E2. Antioxid Redox Signal. 2017;27(8):453‐471. 28006954 10.1089/ars.2016.6800 PMC5564046

