# Supplementary figures and images for: Ellagic acid protects dopamine neurons from rotenone‐induced neurotoxicity via activation of Nrf2 signalling
Source: J Cell Mol Med. 2020 Jul 12;24(16):9446–56. doi: 10.1111/jcmm.15616 (PMC7417702; doi:10.1111/jcmm.15616)

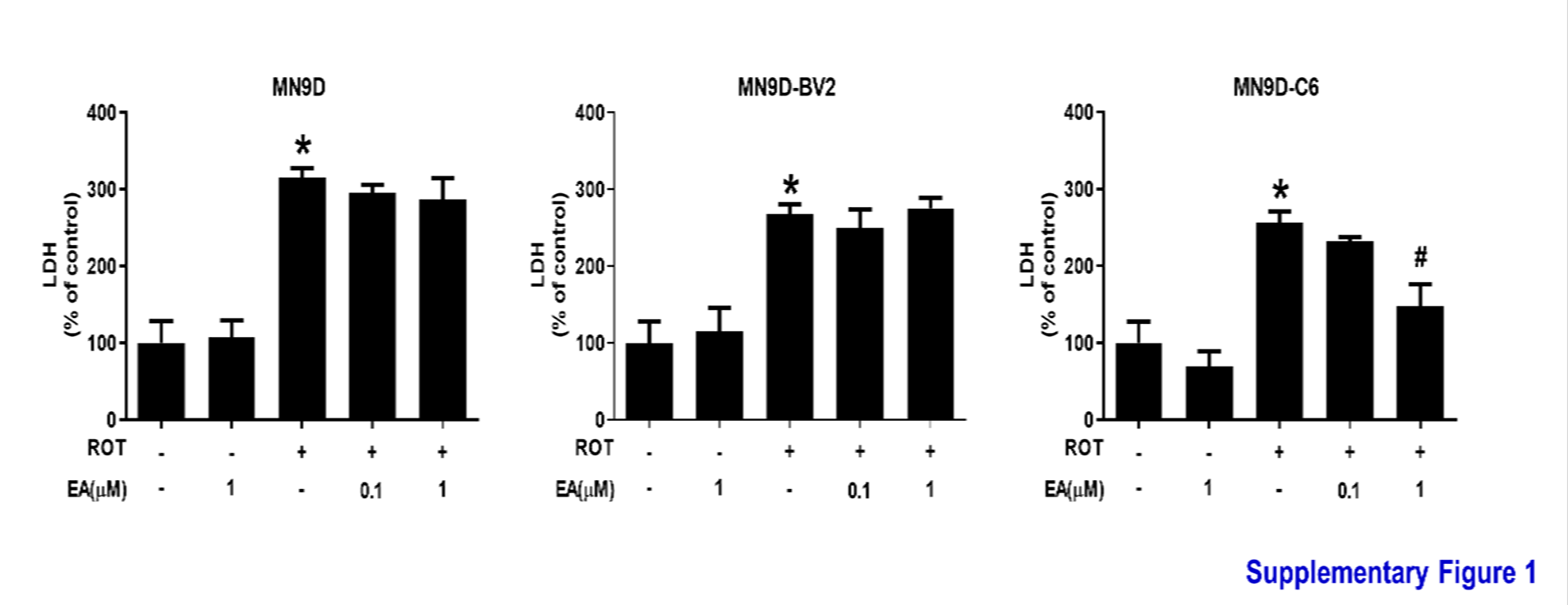

Supplement: Supplementary file 1 — Fig S1 [file JCMM-24-9446-s001.tif]

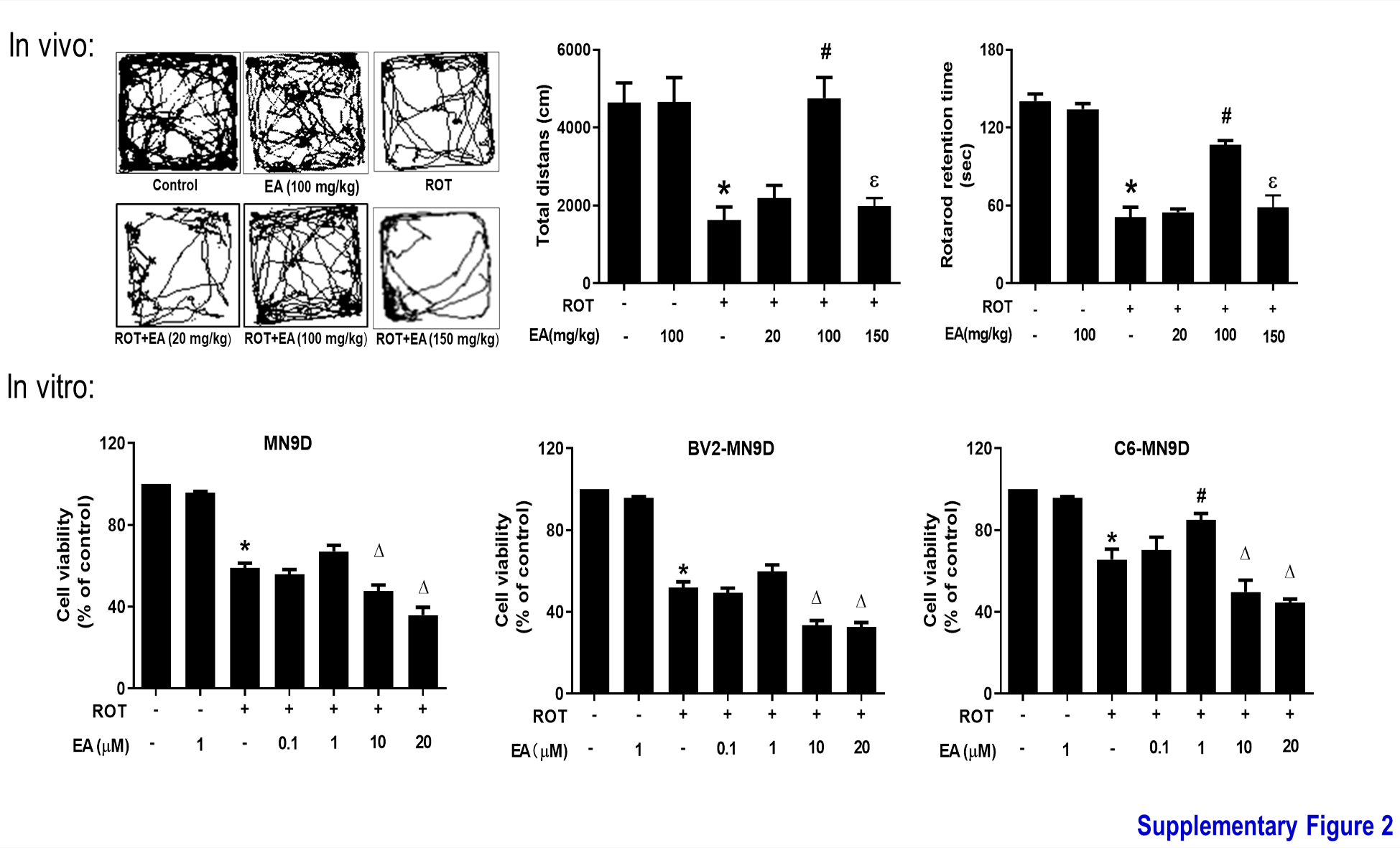

Supplement: Supplementary file 2 — Fig S2 [file JCMM-24-9446-s002.tif]

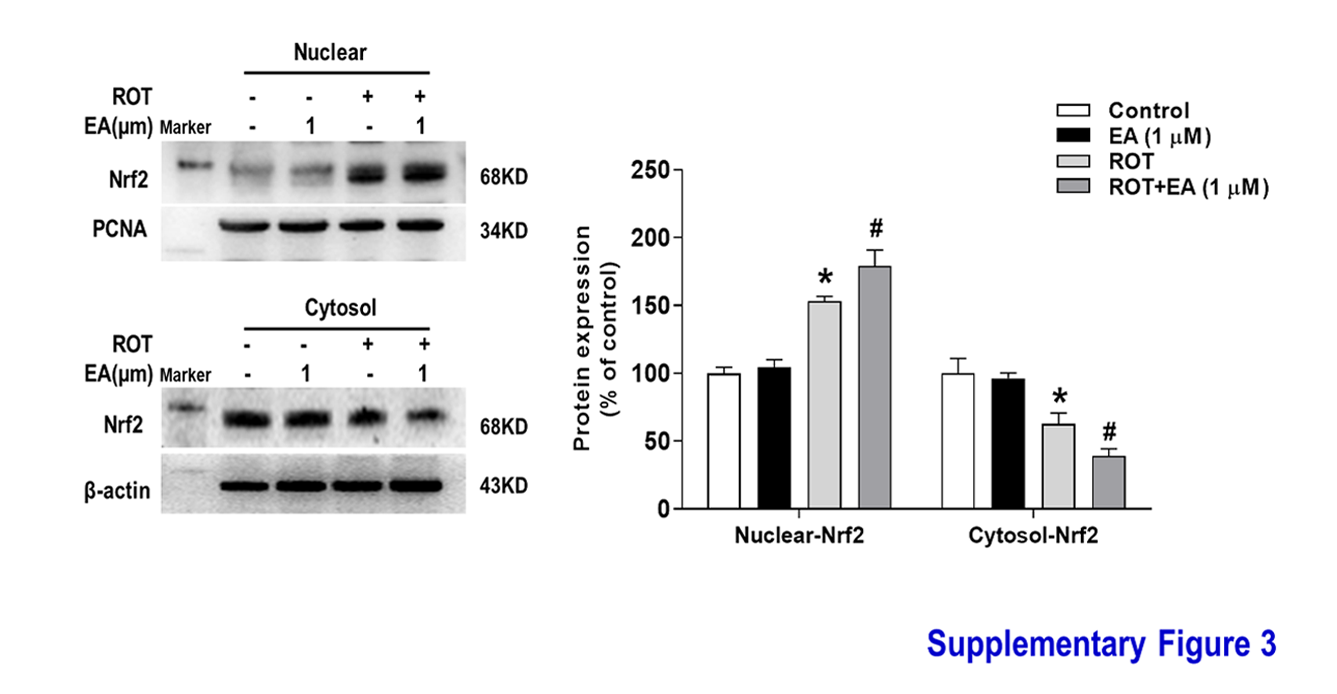

Supplement: Supplementary file 3 — Fig S3 [file JCMM-24-9446-s003.tif]
